# Modifiable Risk Factors for Accelerated Decline in Processing Speed: Results from Three Dutch Population Cohorts

**DOI:** 10.14283/jpad.2023.64

**Published:** 2023-06-04

**Authors:** E. Jaarsma, A. Nooyens, Almar A. L. Kok, S. Köhler, M. van Boxtel, W. M. M. Verschuren, M. Huisman

**Affiliations:** 1 Center for Nutrition, Prevention, and Health Services, National Institute for Public Health and the Environment (RIVM), Bilthoven, the Netherlands; 2 Amsterdam UMC location Vrije Universiteit Amsterdam, Epidemiology and Data Science, De Boelelaan 1117, Amsterdam, Netherlands; 3 Amsterdam Public Health, Aging and Later Life, Amsterdam, the Netherlands; 4 Department of Psychiatry, Amsterdam Public Health, Amsterdam University Medical Center, Vrije Universiteit, Amsterdam, the Netherlands; 5 School for Mental Health & Neuroscience, Faculty of Health, Medicine and Life Sciences, Maastricht University, Maastricht, the Netherlands; 6 Department of Psychiatry and Neuropsychology, Faculty of Health, Medicine and Life Sciences, Maastricht University, Maastricht, the Netherlands; 7 Alzheimer Centrum Limburg, Maastricht University Medical Center+, Maastricht, the Netherlands; 8 Julius Center for Health Sciences and Primary Care, University Medical Center Utrecht, Utrecht University, Utrecht, the Netherlands; 9 Department of Sociology, Faculty of Social Sciences, Vrije Universiteit, Amsterdam, the Netherlands; 10 Amsterdam UMC Locatie De Boelelaan: Amsterdam UMC Locatie VUmc, Amsterdam, the Netherlands

**Keywords:** Cognitive decline, risk factors, processing speed, general population

## Abstract

**Background:**

Several lifestyle, cardiovascular and psychosocial factors are associated with risk of cognitive decline and dementia. We studied the independent associations of a broad set of modifiable risk factors with decline in processing speed in three large population-based cohorts with up to 23 years of follow-up.

**Methods:**

We used data of 9,666 participants from the Doetinchem Cohort Study, the Longitudinal Aging Study Amsterdam, and the Maastricht Aging Study. Decline in processing speed was measured with the letter digit substitution task or the alphabet coding task and modeled using quadratic latent growth curves. Associations of modifiable risk factors with level and rate of decline in processing speed were investigated by estimating associations with level of processing speed at different centering ages.

**Results:**

Latent growth curves showed that decline in processing speed accelerated with age. Smoking, not drinking alcohol and depressive symptoms were associated with a lower level of processing speed in all cohorts. In two of the cohorts, more physical activity, drinking more than two glasses of alcohol per day, higher BMI and diabetes were associated with a lower level of processing speed. Depressive symptoms and diabetes were also associated with faster decline in processing speed.

**Conclusion:**

Several modifiable risk factors are associated with the level of processing speed in older age, while few are also related to the rate of decline.

**Electronic Supplementary Material:**

Supplementary material is available in the online version of this article at 10.14283/jpad.2023.64.

## Introduction

Dementia is often preceded by a period of accelerating cognitive decline, which can be detected years, or even decades, before diagnosis (1, 2). The association between modifiable risk factors and cognitive decline has been studied in several reviews (3, 4). They have consistently concluded that various modifiable risk factors within the lifestyle, cardiovascular and psychosocial domains can increase risk. A recent review concluded that by eliminating 12 specific modifiable risk factors, 40% of dementia cases could be prevented or postponed (4).

Many studies included in these reviews, however, have focused on single or a limited number of risk factors. This is a potential problem, since their effects on cognitive decline may partly overlap. If risk factors are not examined simultaneously, this may lead to potential overestimation of their effects on cognitive decline. Furthermore, studies that did take into account a wider range of risk factors often examined the level of cognitive functioning at only a single follow-up wave, allowing only linear decline to be estimated. This is potentially inaccurate because cognitive decline is expected to accelerate with age. Insight into associations with the rate of decline is also crucial because it can help us identify whether modifiable risk factors have ongoing effects in old age or whether their effects are already determined at a younger age. If the effects would be ongoing or associated with an acceleration in decline, interventions may still be useful in older age.

One of the cognitive domains that is affected in both normal cognitive aging (5) and dementia 6 is processing speed. Decline in processing speed starts relatively early in life (7, 8), is associated with change in other cognitive domains (9), and is hypothesized to be a driver of impairment in other cognitive domains (7). Because of the relatively early start of decline, processing speed is an important domain to look at when investigating risk factors for cognitive decline over the adult life course from midlife onwards.

Several recent studies did investigate the association between a broader set of risk factors and rate of decline in processing speed over 8, 10 and 26 years respectively (10–12). Several modifiable risk factors were associated with the initial level and rate of decline in processing speed in two of these studies (10, 12), while the other did not find any associations with BMI, smoking, alcohol use, cardiovascular disease, hypertension or diabetes (11). Of the studies that did find associations with modifiable risk factors, one reported associations of physical inactivity and depression with initial level of processing speed but not with rate of decline in adults over 50 years old, while current smoking was associated with a higher rate of decline in processing speed. In participants over 60 years old, this association was not found (10). Another study reported an association of smoking, body mass index, and diabetes with a lower level of processing speed at age 43, but no associations with the rate of decline between ages 43 and 69 (12). These findings highlight the importance of investigating both the effects of multiple risk factors as well as examining rates of decline in addition to levels of processing speed.

We investigate the independent effects of modifiable risk factors on the individual differences in initial level and subsequent rate and acceleration of decline in processing speed with aging. We bring together a broad range of modifiable risk factors that have previously been shown to be associated with cognitive functioning in several reviews and meta-analyses (3, 4). Based on this literature, we investigate three main groups of modifiable risk factors: 1) lifestyle and psychosocial factors, 2) cardiovascular factors and 3) cardiometabolic diseases. We are in the unique position to examine robustness of findings using harmonized analyses in three Dutch population-based cohorts from several regions in the country with 12 to 23 years of follow-up in adults with a wide age range.

## Methods

### Cohorts

We used data from three Dutch population-based cohort studies: the Doetinchem Cohort Study (DCS) (13), the Longitudinal Aging Study Amsterdam (LASA) (14) and the Maastricht Aging Study (MAAS) (15). All studies have been described in more detail elsewhere (13–15).

DCS is an ongoing cohort study that started in 1987 with a random sample of 7,768 inhabitants of the Dutch town of Doetinchem aged 20–59 years. Two thirds of the initial sample was invited for follow-up measurement waves, which are conducted every five years. We used data from 1995 onwards, because measurement of cognition started in that year (N = 4,746). Cognitive testing of participants starts in the first measurement round in which they are at least 45 years old and is then repeated every five years. Data was available for up to five repeated measurements over 20 years.

LASA started in 1992 with 3,107 participants aged 55–85 years. In 2002, a second cohort consisting of 1,002 people of 55–64 years old was added. Participants were sampled from three distinct regions, to have a sample that is representative of the Dutch national population. Older people and especially older men were oversampled. Cognitive tests are available for all participants and follow-up is conducted about every three years. We pooled data from the two cohorts, which was available for up to eight waves over 23 years in the first cohort and up to five waves over 13 years in the second cohort (pooled N = 3,767).

Finally, the Maastricht Aging Study started in 1995 with 1,823 participants from the city of Maastricht aged 24–80 years old. Participants were randomly sampled from a patient database of general practices connected to the University of Maastricht, The Netherlands. We only used data from participants aged 50 and older in the first round, because cognitive data was available for four follow-up moments and up to 12 years in this group (N = 1,133).

### Cognitive outcome measure

Two similar cognitive measures were used for assessing processing speed: the Letter-Digit Substitution Task (LDST) in DCS and MAAS (16), and the Alphabet Coding Task (ACT) in LASA (17). These tests mainly measure information processing speed, although they also tap into other cognitive domains, such as attention and working memory. In both tasks, a code linking two sets of characters is shown to the participants at the top of a response sheet. On the next lines, a series of characters is shown. In the LDST, respondents had to write down the corresponding character. In the ACT, respondents had to verbally name the corresponding character.

When using repeated measures of cognitive tests, it is important to consider potential practice effects due to repeated exposure to the same tests (18). We corrected cognitive test scores for repeated testing to minimize learning effects. The correction was performed by fitting a linear mixed model in which dummy variables representing the number of retests predicted the LDST or ACT score. The correction models were adjusted for age at baseline, age at baseline squared, sex and level of education and included random slopes for age. The regression coefficient for the dummy variable represents the part of the test score that can be attributed to having been exposed to the same test a certain number of times and thus is a representation of the overestimation of actual cognitive ability. Therefore, a corrected score was computed by subtracting the regression coefficient for the number of repeated tests from the raw test score.

Because of the difference in tests across cohorts, it was necessary to further harmonize the test scores. This was done using a method previously used by Lipnicki et al. (19). In each cohort, a linear regression model with the cognitive score as the dependent variable and age, sex, and number of years of education as independent variables was applied to baseline data. Scores were then standardized by subtracting the estimated marginal mean at baseline and at fixed values for a common set of covariates (age = 55, sex = 50% male/female, level of education = lower/intermediate general education or lower vocational education) from the raw score at each wave and dividing by the standard deviation of the residuals.

### Risk factors

In addition to demographic and genetic factors, three main groups of modifiable risk factors were distinguished: 1) lifestyle and psychosocial factors, 2) cardiovascular factors and 3) cardiometabolic diseases.

Demographic and genetic factors included sex, level of education and apolipoprotein E ε4 (APOE4) genotype. Level of education was classified into four categories (primary education or less, lower/intermediate general education or lower vocational education, intermediate vocational education or higher general education, and higher vocational education or university). APOE4 genotype was dichotomized into carriers (presence of at least one ε4 allele) and non-carriers.

Lifestyle and psychosocial factors included smoking, alcohol consumption, physical activity, depressive symptoms, sleep duration, healthy nutrition, and social network complexity. Smoking was dichotomized into current non-smokers and current smokers. Alcohol use was classified into three categories: no alcohol use, moderate alcohol use (up to 2 glasses per day) and high alcohol use (more than 2 glasses per day). In DCS and LASA, duration and intensity of physical activity were summarized in MET-hours per week, where 1 metabolic equivalent (MET) is defined as the body’s caloric need during a certain type of physical activity divided by the caloric need during rest. In the case of MAAS, physical activity was measured as the number of active hours per day. Physical activity measures were then standardized into cohort-specific z-scores. Depressive symptoms were assessed with the Mental Health Inventory-5 in DCS (20), the Center for Epidemiologic Studies Depression Scale in LASA (21), and the depression subscale of the Symptom Check List-90 in MAAS (22). In each cohort, the scores on the respective scales were standardized into z-scores. Sleep duration was measured in hours/night and classified into low (< 7 hours), normal (7–9 hours) or high (9 or more hours). This classification was based on recommendations by the American Academy of Sleep Medicine and Sleep Research Society (23). Healthy nutrition was assessed in DCS only and was based on adherence to the Dutch guidelines for a healthy diet. Adherence was measured with the Dutch Healthy Diet Index 2015 (DHD15) (24), which was calculated based on a food frequency questionnaire (25). Because alcohol consumption was used as a separate variable, it was not used in calculating the DHD15. Coffee and salt consumption could not be included either, because the used FFQ does not distinguish between filtered and unfiltered coffee and added salt intake was not assessed. This resulted in a final score ranging from 0–120, with a higher score indicating better adherence to the Dutch guidelines for a healthy diet. Social network complexity was assessed in LASA only and was measured as the number of different social roles a person fulfilled out of 13 options including spouse, child, child-in-law, sibling, sibling-in-law, parent, relative, close friend, acquaintance, neighbor, (former) colleague or group member of a (voluntary) organization (26). In the other cohorts no measures of social network size or complexity were available.

Cardiovascular factors included body mass index (BMI, from objectively measured height and weight and measured in kg/m^2^), systolic blood pressure (mmHg) and total cholesterol (mmol/L). Models that included systolic blood pressure and cholesterol were adjusted for use of antihypertensive and antilipemic drugs.

Finally, cardiometabolic diseases included history of diabetes mellitus, myocardial infarction and/or stroke were obtained through medical records, reports of general practitioners and/or self-report. Models that included stroke were adjusted for incident stroke during follow-up. Not all risk factors were available in each cohort. Availability of risk factors and characteristics of the samples are summarized in Table 2.

### Statistical analyses

We used latent growth curve models within a structural equation modelling framework to analyze the trajectories of processing speed over time. We started out by fitting models to identify the best-fitting shape of the trajectories of cognitive decline. Then we fitted conditional models to identify risk factors associated with the shape of the trajectories.

#### Trajectory shape

The first step in the analyses was to verify that decline in processing speed indeed accelerated with age. Three trajectory shapes were assessed: an intercept-only, a linear and a quadratic model. These models were fitted to the repeated harmonized cognitive scores in each cohort separately. Age was used as the time-variable in the linear and quadratic models. Model fit was assessed using the Bayesian information criterion (BIC), with a lower BIC indicating better model fit.

#### Associations with risk factors

Because several of the modifiable risk factors are in each other’s causal path, we ran a sequence of models that we assume to most closely follow the causal path. Risk factors were added in the following order: (1) lifestyle and psychosocial factors, (2) model 1 + cardiovascular factors, (3) model 2 + cardiometabolic diseases. All conditional models were adjusted for age, sex, level of education and carriage of the APOE4 allele.

Since we found that a quadratic model fitted the data best, interpretation of the associations of risk factors with the trajectories was complex, because the value of the intercept and linear slope parameters depend on the age at which the trajectory is centered, even though the model fit and the modelled trajectories remain the same (27). This is also true for the associations with the intercept and the linear slope. In less technical terms: the association of a risk factor with the course of a trajectory cannot be summarized in one number, so we have to look at multiple associations at the same time to determine whether there is an association of the risk factor with the shape of the trajectory.

Therefore, to correctly interpret the association of the risk factors with the course of the trajectories, we employed a method adopted from Muthén et al. (28). We centered age at three different timepoints, i.e., 55, 65 and 75 years, and used the associations of baseline risk factors with level of processing speed at each of these timepoints to assess whether risk factors were associated with the trajectory of decline. It is important to clarify that we did not do this to identify associations of risk factors with processing speed at those centering ages, but to identify differences in the course of trajectories with ageing that cannot be identified by only looking at one timepoint.

Specifically, if there is no association of a baseline risk factor with the level of processing speed at any of the centering ages, we can conclude that there is no association of the risk factor with the level or rate of decline of processing speed. However, if there is an association of a baseline risk factor with the level of processing speed at one or two centering ages, we can conclude that the rate of decline is different at some point in the trajectory. Furthermore, if associations of similar strength are present at all centering ages, we can conclude that there is an association with the level of processing speed, but not with the rate of decline in processing speed.

Missing data in both the outcome and the covariates were handled in the model by full-information maximum likelihood (FIML). Statistical analyses were performed with the openMx package in R statistical software (29).

## Results

We identified latent growth trajectories for 9,666 individuals from 3 population cohorts. In all cohorts, around half of the participants were female. The average baseline age was 55 years in DCS, 68 years in LASA and 62 years in MAAS. Level of education also differed; the percentage of participants having completed lower vocational education or less was 48% in DCS, 70% in LASA and 33% in MAAS (Table 1).

**Table 1:** Baseline characteristics

	**DCS (N = 4,746)**	**LASA (N = 3,767)**	**MAAS (N = 1,133)**
Processing speed			
LDST/ACT (no. correct (sd))	33.3 (7.2)	21.3	28.8 (7.1)
Demographic and genetic factors			
Age (years (sd))	54.8 (6.6)	67.8 (8.9)	61.8 (10.3)
*Sex (%)*			
Male	47.9	49.8	48.1
Female	52.1	50.2	51.9
*Level of education (%)*			
Primary or less	22.1	38.7	16.4
Lower/intermediate general or lower vocational	26	31.8	16.4
Intermediate vocational or higher general	43.3	15.6	49.3
Higher vocational or university	8.6	13.9	17.9
APOE4 carrier (%)	28.7	28.1	27.2
Lifestyle and psychosocial factors			
Current smoking (%)	23.8	26.5	25.1
*Alcohol (%)*			
No alcohol	31.5	18.7	22.9
1–2 glasses per day	49.6	59	53.5
> 2 glasses per day	18.9	22.3	23.6
*Physical Activity*			
MET-h per week (sd)	104.7 (61.9)	60.3 (45.6)	-
Active hours per day (sd)	-	-	7.8 (3.5)
*Depressive Symptoms*			
MHI-5 (sd)	77.2 (15.2)	-	-
CES-D (sd)	-	8.0 (7.8)	-
SCL-90, depression subscale (sd)	-	-	20.8 (6.2)
*Sleep (%)*			
< 6 hours per night	20	34.1	-
7–9 hours per night	73.5	48.7	
≤ 9 hours per night	6.5	17.2	-
Nutrition (DHD15-score (sd))	66.0 (13.6)	-	-
Social network diversity (no. social roles (sd))	-	4.7 (1.9)	-
Cardiovascular factors			
Body Mass Index (kg/m^2^ (sd))	26.5 (4.0)	27.0 (4.1)	27.6 (4.4)
Systolic blood pressure (mmHg (sd))	130.9 (17.9)	149.4 (25.6)	138.2 (19.8)
Total cholesterol (mmol/L (sd))	5.8 (1.0)	6.3 (1.3)	-
Cardiometabolic diseases			
Diabetes Mellitus (%)	3.0	7.6	5.9
History of myocardial infarction (%)	2.4	8	6.4
History of stroke (%)	1.7	4.7	-

For continuous variables mean(sd), for categorical variables %. LDST = Letter Digit Substitution Task, ACT = Alphabet Coding Task, MET-h = metabolic equivalent hour, MHI-5 = Mental Health Inventory-5, CES-D = Center for Epidemiologic Studies Depression Scale, SCL-90 = Symptom Checklist-90

### Trajectory shape

**Figure 1:**
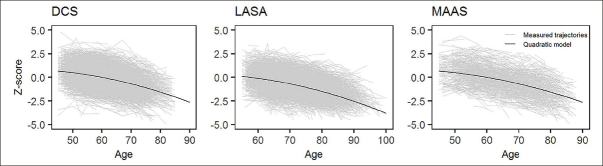
Observed and estimated trajectories of decline in processing speed

We tested intercept-only, linear, and quadratic growth models in each cohort. A quadratic growth model resulted in the lowest BIC in all cohorts and was therefore judged to fit the data best (Table 2). The identified trajectories showed that decline in processing speed accelerates with age (Figure 1). There are no major differences between the cohorts in the average trajectory.

**Table 2:** Model coefficients and model fit for unconditional models

**Cohort**	**Model**	**Intercept**		**Slope**		**Quadratic slope**		
		**Estimate**	**95%CI**	**Estimate**	**95%CI**	**Estimate**	**95%CI**	**BIC**
LASA	Intercept	−0.81	(−0.85; −0.77)	-	-	-	-	−70129.2
LASA	Linear	0.49	(0.44; 0.54)	−0.74	(−0.76; −0.72)	-	-	−75449.8
LASA	Quadratic	0.22	(0.17; 0.28)	−0.35	(−0.40; −0.29)	−0.12	(−0.13; −0.10)	−75772.1
DCS	Intercept	0.07	(0.04; 0.10)	-	-	-	-	−75150.8
DCS	Linear	0.36	(0.33; 0.66)	−0.63	(−0.64; −0.61)	-	-	−80233.5
DCS	Quadratic	0.38	(0.35; 0.41)	−0.5	(−0.53; −0.48)	−0.1	(−0.11; −0.09)	−80469.3
MAAS	Intercept	−0.46	(−0.54; −0.39)	-	-	-	-	−14675.9
MAAS	Linear	0.36	(0.29; 0.44)	−0.73	(−0.77; −0.69)	-	-	−15967.3
MAAS	Quadratic	0.34	(0.26; 0.41)	−0.4	(−0.46; −0.34)	−0.17	(−0.19; −0.14)	−16182.7

CI = confidence interval

### Associations with modifiable risk factors

**Figure 2:**
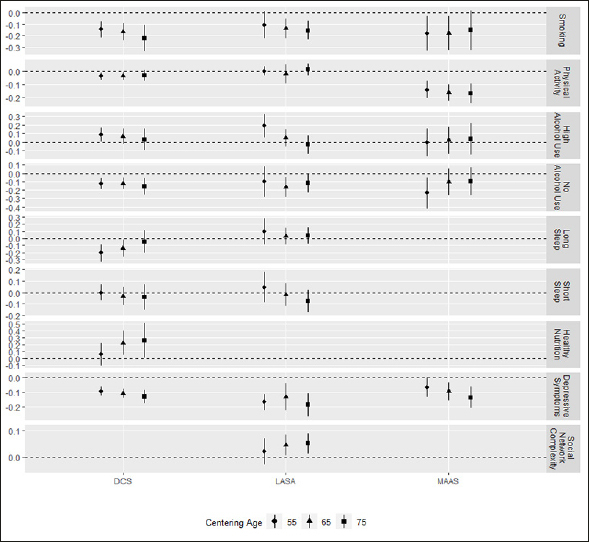
Associations of lifestyle and psychosocial factors with level of processing speed at the different centering ages

**Figure 3:**
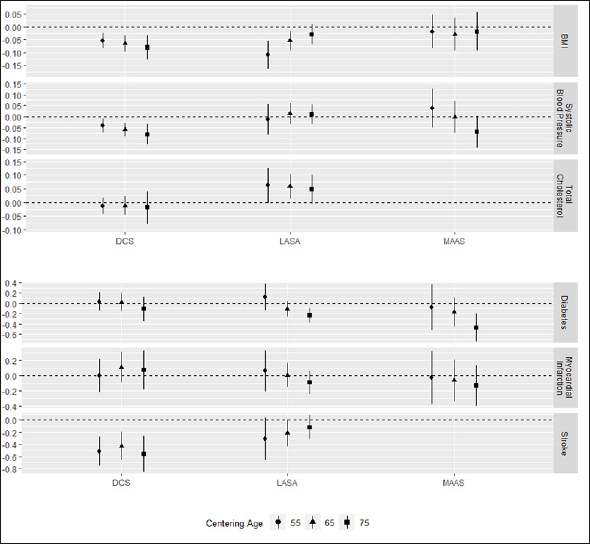
Associations of cardiovascular factors and cardiometabolic disease with level of processing speed at the different centering ages

Associations of modifiable risk factors with the level of cognitive functioning were assessed at three different centering ages. Since associations with risk factors did not differ notably between submodels, only results for the model including all risk factors are presented. All coefficients for continuous variables are standardized. Associations and 95% confidence intervals are shown in Figure 2 and 3, while detailed results can be found in Supplementary Table 1.

Whiskers represent 95% confidence intervals.

Whiskers represent 95% confidence intervals.

### Lifestyle and psychosocial factors

Within the group of lifestyle and psychosocial factors, smoking was associated with level of processing speed in all cohorts at almost all centering ages (age 55/65/75 DCS: β = −0.18 / −0.18 / −0.16; LASA: β = −0.10 / −0.14 /−0.15; MAAS: β = −0.18 / −0.18 / −0.15; statistically significant associations in bold). Depressive symptoms were associated with lower processing speed in all cohorts. In DCS and MAAS, the associations of depressive symptoms with processing speed were more pronounced at older centering ages (age 55/65/75 DCS: β = −0.09 / −0.11 / −0.13; LASA: β = −0.17 / −0.13 /−0.19; MAAS: β = −0.07 / −0.09 / −0.14). This indicates that depressive symptoms may be associated with a faster rate of decline in processing speed as well. For the other lifestyle and psychosocial factors, we found inconsistent results across cohorts. More (intense) physical activity was associated with worse cognitive function in DCS and MAAS (age 55/65/75 DCS: β = −0.04 / −0.04 / −0.03; MAAS: β = −0.14 / −0.16 / −0.17), while we did not find a statistically significant association in LASA. There was no indication of an association with the rate of decline. High alcohol use was associated with better cognitive function at the youngest centering age of 55 years in DCS (β = 0.09) and LASA (β = 0.19). In LASA, the association approached zero at 75 years, indicating that trajectories for high alcohol use and moderate alcohol use converged after the age of 55, but the evidence for this was not consistent across cohorts. No alcohol use was associated with worse cognitive function in all cohorts but at different centering ages. In MAAS, trajectories started at distinct levels and then converged, but we did not find a similar pattern in the other cohorts (age 55/65/75 DCS: β = −0.13 / −0.12 / −0.16; LASA: β = −0.10 / −0.16 / −0.12; MAAS: β = −0.23 / −0.10 /−0.10).

The remaining lifestyle and psychosocial factors could not be investigated in all cohorts. Sleep duration was available in DCS and LASA. Long sleep duration was associated with worse cognition at 55 and 65 years in DCS (age 55/65/75 DCS: β = −0.21 / −0.14 / −0.05), but not in LASA. We found no association between short sleep duration and cognition in either DCS or LASA. Healthy nutrition was only studied in DCS, where we found a positive association between healthy nutrition and better cognitive function at centering ages of 65 and 75 years (age 55/65/75 DCS: β = 0.09 / 0.34 / 0.40), indicating slower decline in those with a healthy diet. Finally, social network complexity was studied in LASA only, where we found that higher social network complexity was associated with better cognition at 65 and 75 years but not at age 55 years (age 55/65/75 LASA: β = 0.02 / 0.04 / 0.05), indicating slower decline in those with high social network complexity.

### Cardiovascular factors

Higher BMI was associated with worse cognition in two out of three cohorts. In DCS we found this association at all centering ages, while we found it only at 55 and 65 years in LASA cohorts (age 55/65/75 DCS: β = −0.05 / −0.07 / −0.08; LASA: β = −0.11 / −0.05 / −0.03; statistically significant associations in bold). In MAAS, we found no association of BMI with cognition. We found conflicting results for the association with rate of decline, since trajectories diverged with age in DCS, while they converged in LASA. Higher systolic blood pressure was associated with worse cognition at all centering ages and with faster decline in DCS (age 55/65/75 DCS: β = −0.04 / −0.07 / −0.08). However, in LASA and MAAS, no associations with systolic blood pressure were found. Finally, total cholesterol was available in DCS and LASA. We found that higher total cholesterol was associated with better cognition at age 55 and 65 in LASA but not in DCS (age 55/65/75 LASA: β = 0.06 / 0.06 / 0.05).

### Cardiometabolic diseases

In LASA and MAAS, diabetes mellitus was associated with worse cognition at 75 years only (age 75 LASA: β = −0.24; MAAS: β = −0.47; statistically significant associations in bold), indicating that participants with diabetes started at similar levels of processing speed as participants without diabetes, but declined faster. In DCS, we found no association between DM and processing speed. Furthermore, we found no statistically significant associations between a history of myocardial infarction and cognition, while history of stroke was associated with worse cognition in DCS only (age 55/65/75 DCS: β = −0.52 / −0.43 / −0.56). No associations with history of stroke were found in LASA, while this could not be studied in MAAS.

## Discussion

### Main findings

We found that the decline in processing speed accelerates with age. Several risk factors were associated with the level of processing speed. We found the most consistent associations for smoking and depressive symptoms with lower processing speed. Furthermore, although not supported consistently in all three cohorts, we found evidence that depressive symptoms and diabetes are associated with the rate of decline in processing speed as well. Healthy nutrition and higher social network complexity were associated with slower rate of decline, but this could only be studied in one of the cohorts.

### Comparison with previous findings

The finding that decline in processing speed accelerates with age is in line with previous research (30–32). Also in line with previous research, only a few of the risk factors that were associated with the level of processing speed also showed an association with the rate of decline (10–12). This might be explained by the two concepts of preserved differentiation and differential preservation, as proposed by Salthouse (33, 34). Preserved differentiation means that differences in processing speed that develop earlier in life are more likely to persist into old age, without becoming wider or narrower. Most of the risk factors we found seem to follow this pattern, with smoking being the most pronounced example across studies. If a risk factor is associated with a pattern of preserved differentiation, there could be consequences for the effectiveness of interventions aimed at this risk factor in later adulthood. These interventions may have to take place earlier in life, i.e., before differences in cognition have been developed.

In contrast, differential preservation means that people differ in the extent to which their cognitive abilities are preserved in later life. Genetic risk factors, health behaviors and psychosocial factors may influence this, and evidence for this would be risk factors that are not only associated with the level but also with the decline in cognitive functioning at older ages. One clear example of a risk factor that has been associated with differential preservation in our study and others is APOE4 (34). We found the same for depressive symptoms and diabetes. Previous studies have also found that diabetes is associated with faster decline in processing speed (34, 35). Depressive symptoms have also previously been associated with cognitive ability. It is still unclear whether depressive symptoms are an actual risk factor for cognitive decline and dementia, or whether they may be part of the prodromal phase of dementia (36, 37). Our finding that depressive symptoms were already associated with the trajectory of decline at an early age and in cohorts with considerable follow-up, suggests that they are not just a symptom of prodromal dementia. In addition, it has been shown previously that midlife depressive symptoms are associated more strongly with vascular dementia than with Alzheimer’s dementia (36). This is a possible explanation for our findings, since processing speed is also associated more strongly with vascular dementia than with Alzheimer’s dementia (38).

Analyses on UK Biobank data and complementary meta-analyses concluded that type 2 diabetes is associated with more impairment in processing speed (39). However, this conclusion was solely based on cross-sectional data, and we provide further evidence that this association also exists longitudinally, in addition to previous similar findings in MAAS (40). The risk factors that are associated with a pattern of differential preservation, like diabetes and potentially depression, could be especially interesting for interventions in later life, since their continual effects suggest that there may be possibilities of slowing the rate of decline even at higher ages.

Finally, we did not find any associations of history of myocardial infarction and processing speed, even though previous studies found an increased risk of vascular dementia in myocardial infarction survivors (41) and faster cognitive decline after incident myocardial infarction or angina (42). As far as we are aware, no studies have looked specifically at the association between history of myocardial infarction and decline in processing speed, so it is possible that other cognitive domains are affected more.

### Strengths and limitations

Strengths of our study include the relatively long follow-up, which enabled us to look at nonlinear trajectories of decline. Additionally, we used three different Dutch cohorts from several regions in the country, which meant we could directly replicate our analyses in three different samples and evaluate robustness of the findings. Finally, we included a broad set of risk factors, to get a clearer view of the independent effects of those risk factors.

Several limitations should be acknowledged. First, both processing speed and part of the modifiable risk factors were measured differently in the different cohorts. Although we applied recent recommendations from the literature (19) to enhance comparability between cohorts, it is not possible to make them fully comparable nor to assess exactly how differences in the measurement instruments affected differences in the results between cohorts. Furthermore, it was not feasible to incorporate time-varying risk factors in the analysis, since not all risk factors were repeatedly measured in all cohorts. To ensure that results were comparable between cohorts, we decided to include only baseline risk factors. This may have influenced the association of our risk factors with the trajectories because a change in risk factor exposure could influence the trajectory of cognitive decline. However, including time-varying risk factors in the analysis would have led to potential bias caused by reversed causation. For several risk factors it is likely that they are influenced by beginning cognitive decline, so including them in the analysis would weaken our assumption that the associations we found are explained by the risk factors influencing cognition. We do however acknowledge that studying time-varying risk factors in relation to cognition is an important topic for future research.

Moreover, risk factors were not measured at the same age for each participant. This means that for certain individuals the centering ages are lower than the age at which the risk factors were measured. Even though we adjusted for baseline age, this makes it difficult to infer potential causal effects of risk factor exposure on trajectories of cognitive decline. However, we still believe our results are valuable, especially since individual level data spanning from middle to late adulthood are scarce. A final limitation is that in general, participants who are ill are less likely to return for follow-up, as are participants with more cognitive impairment (43). This could lead to an underestimation of the association between risk factors and processing speed.

## Conclusion

Decline in processing speed accelerates with age. Several modifiable risk factors are associated with the level of processing speed in older age, while few are also related to the rate of decline. This suggests that to prevent cognitive decline, most of the focus should be on eliminating risk factors in midlife or even earlier.

### Electronic Supplementary Material

Appendix

## Notes

*Ethics approval:* All studies were conducted in line with the Helsinki Declaration. DCS has been approved by the external Medical Ethics Committee of the Netherlands Organization for Applied Scientific Research (first 4 rounds) and the Medical Ethics Committee of the University of Utrecht (later rounds). The LASA study was approved by the medical ethics committee of the VU University Medical Center (IRB numbers: 92/138, 2002/141, 2012/361, and 2016.301). MAAS was approved by the local ethics committee of Maastricht University Medical Centre (MEC05-107).
