# Supplementary material for: Modifiable Risk Factors for Accelerated Decline in Processing Speed: Results from Three Dutch Population Cohorts
Source: J Prev Alzheimers Dis. 2023 Jun 4;11(1):108–16. doi: 10.14283/jpad.2023.64 (PMC10994989; doi:10.14283/jpad.2023.64)
Supplement: Supplementary file 1 — Appendix [file 42414_2023_224_MOESM1_ESM.pdf]

**DCS**	**Model 1**						**Model 2**					
Centering Age	55		65		75		55		65		75	
	Estimate	95%CI	Estimate	95%CI	Estimate	95%CI	Estimate	95%CI	Estimate	95%CI	Estimate	95%CI
**Demographic and genetic factors**												
Age	-0,02	(-0,09 ; 0,05)	-0,01	(-0,06 ; 0,03)	-0,05	(-0,12 ; 0,02)	-0,03	(-0,09 ; 0,03)	-0,03	(-0,08 ; 0,02)	-0,07	(-0,15 ; 0,01)
Sex (female)	**0,39**	**(0,33 ; 0,44)**	**0,37**	**(0,31 ; 0,43)**	**0,37**	**(0,28 ; 0,46)**	**0,46**	**(0,40 ; 0,52)**	**0,42**	**(0,35 ; 0,49)**	**0,43**	**(0,33 ; 0,52)**
Level of education (reference = higher vocational or university)												
Primary or less	**-1,61**	**(-1,73 ; -1,49)**	**-1,55**	**(-1,67 ; -1,42)**	**-1,52**	**(-1,68 ; -1,36)**	**-1,48**	**(-1,60 ; -1,36)**	**-1,41**	**(-1,53 ; -1,28)**	**-1,36**	**(-1,52 ; -1,2)**
Lower/intermediate general or lower vocational	**-0,76**	**(-0,84 ; -0,69)**	**-0,77**	**(-0,85 ; -0,69)**	**-0,75**	**(-0,86 ; -0,64)**	**-0,71**	**(-0,78 ; -0,63)**	**-0,70**	**(-0,78 ; -0,62)**	**-0,67**	**(-0,78 ; -0,55)**
Intermediate vocational or higher general	**-0,33**	**(-0,41 ; -0,25)**	**-0,33**	**(-0,42 ; -0,25)**	**-0,35**	**(-0,48 ; -0,23)**	-0,31	(-0,39 ; -0,23)	-0,31	(-0,4 ; -0,22)	-0,32	(-0,45 ; -0,19)
APOE4 carrier (%)	0,01	(-0,06 ; 0,07)	-0,03	(-0,09 ; 0,04)	**-0,15**	**(-0,24 ; -0,05)**	0,00	(-0,07 ; 0,06)	-0,04	(-0,11 ; 0,03)	**-0,16**	**(-0,25 ; -0,06)**
**Lifestyle and psychosocial factors**												
Current smoking (%)							**-0,13**	**(-0,20 ; -0,06)**	**-0,14**	**(-0,21 ; -0,07)**	**-0,18**	**(-0,29 ; -0,07)**
Alcohol (reference = 1-2 glasses per day)												
No alcohol							**-0,14**	**(-0,20 ; -0,07)**	**-0,14**	**(-0,21 ; -0,07)**	**-0,18**	**(-0,28 ; -0,08)**
> 2 glasses per day							**0,08**	**(0,01 ; 0,16)**	0,07	(-0,01 ; 0,15)	0,04	(-0,08 ; 0,16)
Physical activity							**-0,03**	**(-0,06 ; 0,00)**	0,03	(0,00 ; 0,06)	-0,02	(-0,06 ; 0,02)
Depressive Symptoms							**-0,09**	**(-0,12 ; -0,06)**	**-0,11**	**(-0,14 ; -0,08)**	**-0,14**	**(-0,18 ; -0,09)**
Sleep (reference = 7-9 hours per night)												
< 7 h sleep per night							-0,02	(-0,09 ; 0,05)	-0,05	(-0,12 ; 0,03)	-0,06	(-0,17 ; 0,05)
≥ 9 h sleep per night							**-0,23**	**(-0,35 ; -0,12)**	**-0,18**	**(-0,3 ; -0,05)**	-0,09	(-0,25 ; 0,06)
Healthy nutrition							0,10	(-0,04 ; 0,25)	**0,28**	**(0,12 ; 0,44)**	**0,37**	**(0,14 ; 0,6)**
Network complexity												
**Cardiovascular factors**												
Body mass index (kg/m2)												
Systolic blood pressure (mmHg)												
Total cholesterol (mmol/L)												
**Cardiometabolic diseases**												
Diabetes Mellitus (%)												
History of myocardial infarction (%)												
History of CVA (%)												

**DCS (continued)**	**Model 3**						**Model 4**					
	55		65		75		55		65		75	
	Estimate	95%CI	Estimate	95%CI	Estimate	95%CI	Estimate	95%CI	Estimate	95%CI	Estimate	95%CI
**Demographic and genetic factors**												
Age	0,01	(-0,06 ; 0,08)	0,03	(-0,02 ; 0,08)	0,01	(-0,07 ; 0,08)	0,02	(-0,05 ; 0,09)	0,04	(-0,01 ; 0,09)	0,03	(-0,06 ; 0,12)
Sex (female)	**0,44**	**(0,37 ; 0,5)**	**0,40**	**(0,33 ; 0,46)**	**0,40**	**(0,31 ; 0,5)**	**0,44**	**(0,38 ; 0,50)**	**0,40**	**(0,33 ; 0,46)**	**0,40**	**(0,30 ; 0,49)**
Level of education (reference = higher vocational or university)												
Primary or less	**-1,37**	**(-1,5 ; -1,24)**	**-1,26**	**(-1,4 ; -1,13)**	**-1,17**	**(-1,34 ; -1,00)**	**-1,40**	**(-1,52 ; -1,27)**	**-1,28**	**(-1,41 ; -1,16)**	**-1,19**	**(-1,36 ; -1,02)**
Lower/intermediate general or lower vocational	**-0,65**	**(-0,73 ; -0,57)**	**-0,62**	**(-0,71 ; -0,54)**	**-0,56**	**(-0,68 ; -0,44)**	**-0,66**	**(-0,73 ; -0,58)**	**-0,63**	**(-0,72 ; -0,55)**	**-0,58**	**(-0,69 ; -0,46)**
Intermediate vocational or higher general	**-0,27**	**(-0,36 ; -0,19)**	**-0,27**	**(-0,36 ; -0,18)**	**-0,27**	**(-0,40 ; -0,14)**	**-0,28**	**(-0,36 ; -0,21)**	**-0,28**	**(-0,36 ; -0,19)**	**-0,27**	**(-0,40 ; -0,15)**
APOE4 carrier (%)	0,01	(-0,05 ; 0,08)	-0,02	(-0,09 ; 0,05)	**-0,13**	**(-0,22 ; -0,04)**	0,01	(-0,05 ; 0,08)	-0,02	(-0,09 ; 0,05)	**-0,13**	**(-0,23 ; -0,04)**
**Lifestyle and psychosocial factors**												
Current smoking (%)	**-0,14**	**(-0,21 ; -0,07)**	**-0,16**	**(-0,24 ; -0,09)**	**-0,22**	**(-0,33 ; -0,1)**	**-0,15**	**(-0,21 ; -0,08)**	**-0,16**	**(-0,24 ; -0,09)**	**-0,22**	**(-0,33 ; -0,11)**
Alcohol (reference = 1-2 glasses per day)												
No alcohol	**-0,12**	**(-0,19 ; -0,06)**	**-0,12**	**(-0,19 ; -0,05)**	**-0,16**	**(-0,26 ; -0,06)**	**-0,13**	**(-0,19 ; -0,06)**	**-0,12**	**(-0,19 ; -0,05)**	**-0,16**	**(-0,25 ; -0,06)**
> 2 glasses per day	**0,10**	**(0,02 ; 0,18)**	0,08	(-0,01 ; 0,17)	0,05	(-0,07 ; 0,17)	**0,09**	**(0,01 ; 0,17)**	0,07	(-0,02 ; 0,15)	0,03	(-0,09 ; 0,16)
Physical activity	-0,04	(-0,07 ; -0,01)	**-0,04**	**(-0,07 ; -0,01)**	-0,03	(-0,08 ; 0,01)	**-0,04**	**(-0,06;-0,01)**	**-0,04**	**(-0,07;-0,01)**	-0,03	(-0,07;0,01)
Depressive Symptoms	**-0,09**	**(-0,12 ; -0,06)**	**-0,11**	**(-0,14 ; -0,08)**	**-0,13**	**(-0,18 ; -0,09)**	**-0,09**	**(-0,06 ; -0,12)**	**-0,11**	**(-0,07 ; -0,14)**	**-0,13**	**(-0,08 ; -0,17)**
Sleep (reference = 7-9 hours per night)												
< 7 h sleep per night	0,00	(-0,07 ; 0,07)	-0,03	(-0,11 ; 0,05)	-0,04	(-0,15 ; 0,08)	0,00	(-0,07 ; 0,07)	-0,03	(-0,11 ; 0,04)	-0,04	(-0,15 ; 0,07)
≥ 9 h sleep per night	**-0,20**	**(-0,32 ; -0,08)**	**-0,13**	**(-0,26 ; -0,01)**	-0,04	(-0,2 ; 0,12)	**-0,21**	**(-0,33 ; -0,09)**	**-0,14**	**(-0,26 ; -0,02)**	-0,05	(-0,20 ; 0,11)
Healthy nutrition	0,09	(-0,08 ; 0,27)	**0,26**	**(0,08 ; 0,45)**	**0,32**	**(0,06 ; 0,58)**	0,06	(-0,11 ; 0,23)	**0,23**	**(0,05 ; 0,40)**	**0,26**	**(0,01 ; 0,51)**
Network complexity												
**Cardiovascular factors**												
Body mass index (kg/m2)	**-0,05**	**(-0,08 ; -0,02)**	**-0,06**	**(-0,09 ; -0,03)**	**-0,08**	**(-0,12 ; -0,03)**	**-0,05**	**(-0,08 ; -0,02)**	**-0,07**	**(-0,1 ; -0,03)**	**-0,08**	**(-0,13 ; -0,03)**
Systolic blood pressure (mmHg)	**-0,04**	**(-0,07 ; -0,01)**	**-0,06**	**(-0,09 ; -0,03)**	**-0,08**	**(-0,13 ; -0,04)**	**-0,04**	**(-0,07 ; -0,01)**	**-0,06**	**(-0,09 ; -0,03)**	**-0,08**	**(-0,13 ; -0,03)**
Total cholesterol (mmol/L)	-0,02	(-0,05 ; 0,02)	-0,01	(-0,05 ; 0,03)	-0,01	(-0,06 ; 0,04)	-0,01	(-0,04 ; 0,02)	-0,01	(-0,05 ; 0,02)	-0,02	(-0,08 ; 0,04)
**Cardiometabolic diseases**												
Diabetes Mellitus (%)							0,03	(-0,15 ; 0,22)	0,02	(-0,17 ; 0,20)	-0,11	(-0,35 ; 0,13)
History of myocardial infarction (%)							0,00	(-0,22 ; 0,22)	0,11	(-0,10 ; 0,31)	0,07	(-0,19 ; 0,33)
History of CVA (%)							**-0,52**	**(-0,76 ; -0,27)**	**-0,43**	**(-0,66 ; -0,20)**	**-0,56**	**(-0,85 ; -0,26)**

LASA	Model 1						Model 2					
Centering Age	55		65		75		55		65		75	
	Estimate	95%CI	Estimate	95%CI	Estimate	95%CI	Estimate	95%CI	Estimate	95%CI	Estimate	95%CI
**Demographic and genetic factors**												
Age	-0,05	(-0,28 ; 0,18)	-0,03	(-0,12 ; 0,05)	-0,02	(-0,06 ; 0,03)	-0,02	(-0,15 ; 0,10)	0,02	(-0,08 ; 0,12)	0,03	(-0,02 ; 0,08)
Sex (female)	**0,30**	**(0,19 ; 0,42)**	**0,28**	**(0,21 ; 0,35)**	**0,28**	**(0,21 ; 0,35)**	**0,36**	**(0,24 ; 0,47)**	**0,33**	**(0,25 ; 0,41)**	**0,32**	**(0,24 ; 0,40)**
Level of education (reference = higher vocational or university)												
Primary or less	**1,24**	**(1,08 ; 1,41)**	**1,28**	**(1,17 ; 1,39)**	**1,24**	**(1,13 ; 1,35)**	**-1,09**	**(-1,26 ; -0,93)**	**-1,21**	**(-1,32 ; -1,09)**	**-1,18**	**(-1,29 ; -1,07)**
Lower/intermediate general or lower vocational	**0,83**	**(0,66 ; 0,99)**	**0,90**	**(0,79 ; 1,01)**	**0,87**	**(0,77 ; 0,98)**	**-0,56**	**(-0,72 ; -0,41)**	**-0,66**	**(-0,77 ; -0,55)**	**-0,65**	**(-0,76 ; -0,55)**
Intermediate vocational or higher general	**0,61**	**(0,47 ; 0,75)**	**0,61**	**(0,53 ; 0,7)**	**0,58**	**(0,5 ; 0,66)**	**-0,34**	**(-0,52 ; -0,17)**	**-0,39**	**(-0,51 ; -0,27)**	**-0,39**	**(-0,51 ; -0,27)**
APOE4 carrier (%)	0,05	(-0,08 ; 0,19)	0,01	(-0,07 ; 0,1)	**-0,10**	**(-0,18 ; -0,02)**	0,05	(-0,08 ; 0,18)	0,01	(-0,07 ; 0,10)	**-0,12**	**(-0,20 ; -0,03)**
**Lifestyle and psychosocial factors**												
Current smoking (%)							-0,08	(-0,19 ; 0,04)	**-0,13**	**(-0,21 ; -0,05)**	**-0,16**	**(-0,25 ; -0,08)**
Alcohol (reference = 1-2 glasses per day)												
No alcohol							-0,11	(-0,3 ; 0,07)	**-0,29**	**(-0,4 ; -0,19)**	**-0,29**	**(-0,39 ; -0,19)**
> 2 glasses per day							**0,15**	**(0,01 ; 0,28)**	-0,01	(-0,11 ; 0,09)	-0,09	(-0,2 ; 0,02)
Physical activity							0,01	(-0,02 ; 0,05)	**-0,07**	**(-0,12 ; -0,02)**	**-0,07**	**(-0,11 ; -0,03)**
Depressive Symptoms							**-0,17**	**(-0,24 ; -0,11)**	**-0,11**	**(-0,19 ; -0,04)**	**-0,14**	**(-0,21 ; -0,07)**
Sleep (reference = 7-9 hours per night)												
< 7 h sleep per night							0,03	(-0,11 ; 0,16)	-0,05	(-0,15 ; 0,05)	**-0,11**	**(-0,21 ; 0,00)**
≥ 9 h sleep per night							0,08	(-0,11 ; 0,27)	-0,03	(-0,14 ; 0,08)	-0,04	(-0,14 ; 0,07)
Healthy nutrition												
Network complexity							0,02	(-0,03 ; 0,07)	0,03	(0,00 ; 0,07)	0,03	(0,00 ; 0,07)
**Cardiovascular factors**												
Body mass index (kg/m2)												
Systolic blood pressure (mmHg)												
Total cholesterol (mmol/L)												
**Cardiometabolic diseases**												
Diabetes Mellitus (%)												
History of myocardial infarction (%)												
History of CVA (%)												

**LASA (Continued)**	**Model 3**						**Model 4**					
	55		65		75		55		65		75	
	Estimate	95%CI	Estimate	95%CI	Estimate	95%CI	Estimate	95%CI	Estimate	95%CI	Estimate	95%CI
**Demographic and genetic factors**												
Age	-0,03	(-0,23 ; 0,18)	-0,03	(-0,1 ; 0,04)	-0,04	(-0,1 ; 0,01)	-0,01	(-0,14 ; 0,12)	-0,02	(-0,1 ; 0,07)	-0,02	(-0,08 ; 0,04)
Sex (female)	**0,34**	**(0,22 ; 0,46)**	**0,29**	**(0,21 ; 0,36)**	**0,27**	**(0,2 ; 0,35)**	**0,34**	**(0,23 ; 0,46)**	**0,28**	**(0,2 ; 0,36)**	**0,26**	**(0,18 ; 0,34)**
Level of education (reference = higher vocational or university)												
Primary or less	**-1,05**	**(-1,23 ; -0,88)**	**-1,15**	**(-1,27 ; -1,04)**	**-1,11**	**(-1,22 ; -0,99)**	**-1,05**	**(-1,22 ; -0,89)**	**-1,15**	**(-1,27 ; -1,03)**	**-1,11**	**(-1,23 ; -0,99)**
Lower/intermediate general or lower vocational	**-0,54**	**(-0,7 ; -0,38)**	**-0,62**	**(-0,73 ; -0,51)**	**-0,60**	**(-0,71 ; -0,49)**	**-0,54**	**(-0,69 ; -0,39)**	**-0,62**	**(-0,73 ; -0,51)**	**-0,60**	**(-0,71 ; -0,49)**
Intermediate vocational or higher general	**-0,33**	**(-0,5 ; -0,16)**	**-0,35**	**(-0,47 ; -0,22)**	**-0,33**	**(-0,45 ; -0,2)**	**-0,33**	**(-0,5 ; -0,16)**	**-0,34**	**(-0,47 ; -0,22)**	**-0,33**	**(-0,45 ; -0,2)**
APOE4 carrier (%)	0,03	(-0,1 ; 0,17)	0,00	(-0,09 ; 0,09)	**-0,12**	**(-0,2 ; -0,03)**	0,04	(-0,09 ; 0,17)	0,00	(-0,09 ; 0,08)	**-0,12**	**(-0,21 ; -0,04)**
**Lifestyle and psychosocial factors**												
Current smoking (%)	-0,11	(-0,23 ; 0,02)	**-0,14**	**(-0,22 ; -0,06)**	**-0,15**	**(-0,24 ; -0,07)**	-0,10	(-0,22 ; 0,01)	**-0,14**	**(-0,22 ; -0,05)**	**-0,15**	**(-0,24 ; -0,07)**
Alcohol (reference = 1-2 glasses per day)												
No alcohol	-0,11	(-0,30 ; 0,07)	**-0,18**	**(-0,29 ; -0,07)**	**-0,14**	**(-0,25 ; -0,02)**	-0,10	(-0,28 ; 0,08)	**-0,16**	**(-0,28 ; -0,04)**	**-0,12**	**(-0,23 ; 0)**
> 2 glasses per day	**0,18**	**(0,04 ; 0,32)**	0,05	(-0,05 ; 0,14)	-0,02	(-0,13 ; 0,08)	**0,19**	**(0,05 ; 0,32)**	0,05	(-0,05 ; 0,15)	-0,03	(-0,14 ; 0,08)
Physical activity	0,00	(-0,04 ; 0,04)	-0,02	(-0,05 ; 0,01)	0,02	(-0,01 ; 0,06)	0,00	(-0,03 ; 0,04)	-0,02	(-0,09 ; 0,06)	0,02	(-0,03 ; 0,06)
Depressive Symptoms	**-0,17**	**(-0,24 ; -0,09)**	**-0,13**	**(-0,18 ; -0,08)**	**-0,19**	**(-0,25 ; -0,12)**	**-0,17**	**(-0,22 ; -0,11)**	**-0,13**	**(-0,22 ; -0,04)**	**-0,19**	**(-0,26 ; -0,11)**
Sleep (reference = 7-9 hours per night)												
< 7 h sleep per night	0,04	(-0,1 ; 0,18)	-0,02	(-0,11 ; 0,07)	-0,08	(-0,18 ; 0,02)	0,04	(-0,09 ; 0,18)	-0,02	(-0,12 ; 0,08)	-0,08	(-0,17 ; 0,02)
≥ 9 h sleep per night	0,09	(-0,1 ; 0,27)	0,02	(-0,1 ; 0,13)	0,03	(-0,08 ; 0,15)	0,10	(-0,08 ; 0,28)	0,03	(-0,09 ; 0,15)	0,04	(-0,08 ; 0,16)
Healthy nutrition												
Network complexity	0,02	(-0,04 ; 0,08)	**0,05**	**(0,01 ; 0,08)**	**0,05**	**(0,01 ; 0,09)**	0,02	(-0,03 ; 0,07)	**0,04**	**(0,01 ; 0,08)**	**0,05**	**(0,01 ; 0,09)**
**Cardiovascular factors**												
Body mass index (kg/m2)	**-0,11**	**(-0,17 ; -0,05)**	**-0,06**	**(-0,09 ; -0,02)**	-0,04	(-0,07 ; 0,00)	**-0,11**	**(-0,17 ; -0,05)**	**-0,05**	**(-0,09 ; -0,02)**	-0,03	(-0,07 ; 0,01)
Systolic blood pressure (mmHg)	-0,01	(-0,09 ; 0,06)	0,01	(-0,03 ; 0,05)	0,01	(-0,04 ; 0,05)	-0,01	(-0,08 ; 0,06)	0,01	(-0,03 ; 0,06)	0,01	(-0,04 ; 0,05)
Total cholesterol (mmol/L)	**0,07**	**(0,01 ; 0,14)**	**0,07**	**(0,02 ; 0,11)**	**0,06**	**(0,01 ; 0,11)**	0,47	(-0,03 ; 0,97)	**0,45**	**(0,09 ; 0,8)**	0,36	(-0,05 ; 0,78)
**Cardiometabolic diseases**												
Diabetes Mellitus (%)							0,12	(-0,13 ; 0,38)	-0,11	(-0,27 ; 0,05)	**-0,24**	**(-0,38 ; -0,09)**
History of myocardial infarction (%)							0,06	(-0,21 ; 0,33)	0,00	(-0,16 ; 0,16)	-0,09	(-0,25 ; 0,06)
History of CVA (%)							-0,31	(-0,66 ; 0,04)	-0,21	(-0,43 ; 0,01)	-0,12	(-0,32 ; 0,09)

MAAS	Model 1						Model 2					
Centering Age	55		65		75		55		65		75	
	Estimate	95%CI	Estimate	95%CI	Estimate	95%CI	Estimate	95%CI	Estimate	95%CI	Estimate	95%CI
**Demographic and genetic factors**												
Age	**-0,33**	**(-0,49 – -0,18)**	0,00	(-0,07 – 0,08)	**0,20**	**(0,1 – 0,31)**	**-0,36**	**(-0,52 – -0,21)**	-0,03	(-0,11 – 0,05)	**0,17**	**(0,06 – 0,28)**
Sex (female)	-0,05	(-0,19 – 0,09)	-0,06	(-0,19 – 0,06)	0,01	(-0,13 – 0,15)	0,01	(-0,13 – 0,15)	-0,01	(-0,14 – 0,12)	0,07	(-0,07 – 0,22)
Level of education (reference = higher vocational or university)												
Primary or less	**-1,47**	**(-1,74 – -1,19)**	**-1,46**	**(-1,69 – -1,24)**	**-1,30**	**(-1,54 – -1,06)**	**-1,34**	**(-1,62 – -1,06)**	**-1,35**	**(-1,57 – -1,13)**	**-1,20**	**(-1,44 – -0,97)**
Lower/intermediate general or lower vocational	**-0,92**	**(-1,11 – -0,73)**	**-0,87**	**(-1,05 – -0,68)**	**-0,78**	**(-0,98 – -0,57)**	**-0,82**	**(-1,01 – -0,63)**	**-0,78**	**(-0,96 – -0,6)**	**-0,71**	**(-0,91 – -0,51)**
Intermediate vocational or higher general	**-0,53**	**(-0,75 – -0,30)**	**-0,42**	**(-0,64 – -0,20)**	-0,21	(-0,46 – 0,04)	**-0,49**	**(-0,72 – -0,27)**	**-0,39**	**(-0,6 – -0,18)**	-0,21	(-0,46 – 0,04)
APOE4 carrier (%)	-0,11	(-0,29 – 0,07)	-0,09	(-0,26 – 0,09)	-0,10	(-0,30 – 0,10)	-0,12	(-0,3 – 0,07)	-0,10	(-0,27 – 0,07)	-0,10	(-0,3 – 0,1)
**Lifestyle and psychosocial factors**												
Current smoking (%)							**-0,17**	**(-0,33 – -0,02)**	**-0,16**	**(-0,31 – -0,02)**	-0,13	(-0,30 – 0,05)
Alcohol (reference = 1-2 glasses per day)												
No alcohol							**-0,25**	**(-0,44 – -0,07)**	-0,13	(-0,29 – 0,03)	-0,14	(-0,31 – 0,02)
> 2 glasses per day							-0,01	(-0,17 – 0,16)	0,01	(-0,15 – 0,16)	0,01	(-0,18 – 0,19)
Physical activity							**-0,14**	**(-0,20 – -0,07)**	**-0,16**	**(-0,22 – -0,09)**	**-0,16**	**(-0,23 – -0,08)**
Depressive Symptoms							**-0,07**	**(-0,14 – 0,00)**	**-0,10**	**(-0,16 – -0,03)**	**-0,14**	**(-0,22 – -0,07)**
Sleep (reference = 7-9 hours per night)												
< 7 h sleep per night												
≥ 9 h sleep per night												
Healthy nutrition												
Network complexity												
**Cardiovascular factors**												
Body mass index (kg/m2)												
Systolic blood pressure (mmHg)												
Total cholesterol (mmol/L)												
**Cardiometabolic diseases**												
Diabetes Mellitus (%)												
History of myocardial infarction (%)												
History of CVA (%)												

**MAAS (continued)**	**Model 3**						**Model 4**					
	55		65		75		55		65		75	
	Estimate	95%CI	Estimate	95%CI	Estimate	95%CI	Estimate	95%CI	Estimate	95%CI	Estimate	95%CI
**Demographic and genetic factors**												
Age	**-0,37**	**(-0,53 – -0,22)**	-0,03	(-0,11 – 0,06)	**0,20**	**(0,09 – 0,32)**	**-0,38**	**(-0,54 – -0,22)**	-0,02	(-0,10 – 0,07)	**0,23**	**(0,12 – 0,34)**
Sex (female)	0,02	(-0,12 – 0,16)	-0,01	(-0,14 – 0,13)	0,08	(-0,07 – 0,22)	0,02	(-0,13 – 0,17)	-0,01	(-0,15 – 0,12)	0,05	(-0,10 – 0,19)
Level of education (reference = higher vocational or university)												
Primary or less	**-1,33**	**(-1,61 – -1,05)**	**-1,34**	**(-1,57 – -1,12)**	**-1,20**	**(-1,43 – -0,97)**	**-1,32**	**(-1,61 – -1,04)**	**-1,34**	**(-1,57 – -1,12)**	**-1,21**	**(-1,44 – -0,98)**
Lower/intermediate general or lower vocational	**-0,82**	**(-1,01 – -0,63)**	**-0,77**	**(-0,95 – -0,59)**	**-0,69**	**(-0,89 – -0,49)**	**-0,83**	**(-1,02 – -0,64)**	**-0,77**	**(-0,95 – -0,59)**	**-0,69**	**(-0,89 – -0,49)**
Intermediate vocational or higher general	**-0,50**	**(-0,72 – -0,28)**	**-0,39**	**(-0,6 – -0,17)**	-0,19	(-0,43 – 0,06)	**-0,50**	**(-0,72 – -0,28)**	**-0,39**	**(-0,60 – -0,17)**	-0,20	(-0,44 – 0,04)
APOE4 carrier (%)	-0,13	(-0,31 – 0,06)	-0,09	(-0,27 – 0,08)	-0,06	(-0,27 – 0,14)	-0,14	(-0,33 – 0,05)	-0,10	(-0,28 – 0,08)	-0,05	(-0,25 – 0,15)
**Lifestyle and psychosocial factors**												
Current smoking (%)	**-0,18**	**(-0,33 – -0,03)**	-0,17	(-0,32 – -0,03)	-0,14	(-0,31 – 0,04)	**-0,18**	**(-0,33 – -0,03)**	**-0,18**	**(-0,32 – -0,03)**	-0,15	(-0,32 – 0,02)
Alcohol (reference = 1-2 glasses per day)												
No alcohol	**-0,24**	**(-0,42 – -0,05)**	-0,12	(-0,28 – 0,04)	-0,13	(-0,30 – 0,03)	**-0,23**	**(-0,42 – -0,05)**	-0,10	(-0,26 – 0,06)	-0,10	(-0,26 – 0,07)
> 2 glasses per day	0,00	(-0,16 – 0,16)	0,02	(-0,14 – 0,17)	0,03	(-0,16 – 0,21)	0,00	(-0,17 – 0,16)	0,02	(-0,14 – 0,18)	0,04	(-0,15 – 0,22)
Physical activity	**-0,14**	**(-0,2 – -0,07)**	**-0,16**	**(-0,22 – -0,09)**	**-0,17**	**(-0,24 – -0,09)**	**-0,14**	**(-0,21;-0,07)**	**-0,16**	**(-0,23;-0,1)**	**-0,17**	**(-0,24;-0,1)**
Depressive Symptoms	-0,07	(-0,13 – 0,00)	**-0,10**	**(-0,16 – -0,03)**	**-0,14**	**(-0,21 – -0,06)**	-0,07	(-0,13 – 0,00)	**-0,09**	**(-0,16 – -0,03)**	**-0,14**	**(-0,21 – -0,06)**
Sleep (reference = 7-9 hours per night)												
< 7 h sleep per night												
≥ 9 h sleep per night												
Healthy nutrition												
Network complexity												
**Cardiovascular factors**												
Body mass index (kg/m2)	-0,02	(-0,09 – 0,04)	-0,03	(-0,10 – 0,03)	-0,03	(-0,10 – 0,04)	-0,04	(-0,19 – 0,11)	-0,07	(-0,21 – 0,08)	-0,04	(-0,21 – 0,13)
Systolic blood pressure (mmHg)	0,04	(-0,04 – 0,13)	-0,01	(-0,08 – 0,07)	-0,09	**(-0,16 – -0,02)**	0,19	(-0,25 – 0,64)	-0,01	(-0,38 – 0,36)	-0,35	(-0,72 – 0,01)
Total cholesterol (mmol/L)												
**Cardiometabolic diseases**												
Diabetes Mellitus (%)							-0,08	(-0,52 – 0,37)	-0,18	(-0,46 – 0,1)	**-0,47**	**(-0,74 – -0,2)**
History of myocardial infarction (%)							-0,03	(-0,38 – 0,32)	-0,06	(-0,33 – 0,2)	-0,13	(-0,4 – 0,13)
History of CVA (%)												
